# Short-duration dynamic [^18^F]DCFPyL PET and CT perfusion imaging to localize dominant intraprostatic lesions in prostate cancer: validation against digital histopathology and comparison to [^18^F]DCFPyL PET/MR at 120 minutes

**DOI:** 10.1186/s13550-021-00844-0

**Published:** 2021-10-15

**Authors:** Dae-Myoung Yang, Ryan Alfano, Glenn Bauman, Jonathan D. Thiessen, Joseph Chin, Stephen Pautler, Madeleine Moussa, Jose A. Gomez, Irina Rachinsky, Mena Gaed, Kevin J. Chung, Aaron Ward, Ting-Yim Lee

**Affiliations:** 1 Department of Medical Biophysics, The University of Western Ontario, London, ON Canada; 2 Robarts Research Institute, The University of Western Ontario, London, ON Canada; 3 Imaging Program, Lawson Health Research Institute, 750 Base Line Road E, London, ON N6C 2R5 Canada; 4 London Health Sciences Centre, London, ON Canada; 5 Baines Imaging Research Laboratory, London, ON Canada; 6 Department of Oncology, The University of Western Ontario, London, ON Canada; 7 Department of Surgery, The University of Western Ontario, London, ON Canada; 8 St. Joseph’ Health Care, London, ON Canada; 9 Department of Medical Imaging, The University of Western Ontario, London, ON Canada; 10 Department of Pathology and Laboratory Medicine, The University of Western Ontario, London, ON Canada; 11 Department of Radiation Oncology, Fox Chase Cancer Center, 333 Cottman Ave, Philadelphia, PA 19111 USA

**Keywords:** [^18^F]DCFPyL, Prostate-specific membrane antigen (PSMA), CT perfusion, Tracer kinetic modelling, Dominant intraprostatic lesion (DIL)

## Abstract

**Purpose:**

Localized prostate cancer (PCa) in patients is characterized by a dominant focus in the gland (dominant intraprostatic lesion, DIL). Accurate DIL identification may enable more accurate diagnosis and therapy through more precise targeting of biopsy, radiotherapy and focal ablative therapies. The goal of this study is to validate the performance of [^18^F]DCFPyL PET and CT perfusion (CTP) for detecting and localizing DIL against digital histopathological images.

**Methods:**

Multi-modality image sets: in vivo T2-weighted (T2w)-MRI, 22-min dynamic [^18^F]DCFPyL PET/CT, CTP, and 2-h post-injection PET/MR were acquired in patients prior to radical prostatectomy. The explanted gland with implanted fiducial markers was imaged with T2w-MRI. All images were co-registered to the pathologist-annotated digital images of whole-mount mid-gland histology sections using fiducial markers and anatomical landmarks. Regions of interest encompassing DIL and non-DIL tissue were drawn on the digital histopathological images and superimposed on PET and CTP parametric maps. Logistic regression with backward elimination of parameters was used to select the most sensitive parameter set to distinguish DIL from non-DIL voxels. Leave-one-patient-out cross-validation was performed to determine diagnostic performance.

**Results:**

[^18^F]DCFPyL PET and CTP parametric maps of 15 patients were analyzed. SUV_Late_ and a model combining K_i_ and k_4_ of [^18^F]DCFPyL achieved the most accurate performance distinguishing DIL from non-DIL voxels. Both detection models achieved an AUC of 0.90 and an error rate of < 10%. Compared to digital histopathology, the detected DILs had a mean dice similarity coefficient of 0.8 for the K_i_ and k_4_ model and 0.7 for SUV_Late_.

**Conclusions:**

We have validated using co-registered digital histopathological images that parameters from kinetic analysis of 22-min dynamic [^18^F]DCFPyL PET can accurately localize DILs in PCa for targeting of biopsy, radiotherapy, and focal ablative therapies. Short-duration dynamic [^18^F]DCFPyL PET was not inferior to SUV_Late_ in this diagnostic task.

*Clinical trial registration number*: NCT04009174 (ClinicalTrials.gov).

## Introduction

Localized prostate cancer (PCa) is frequently a multifocal disease [1, 2]. It has been reported in 78% of all cases of radical prostatectomy specimens [3]. The dominant intraprostatic lesion (DIL) is the most prominent cancerous lesion that is the largest in size within the prostate. Identifying DIL is clinically important. The size of DIL showed a strong correlation with prostate-specific antigen (PSA) level and biochemical relapse [4, 5]. DIL is often the site where local recurrence and metastasis originates after treatment [6, 7]. Because of these DIL attributes, radiation therapy involving escalated or boosted doses to DIL has been investigated in several randomized control trials and demonstrated improved biochemical recurrence-free survival [8, 9]. In addition to guiding treatment, DIL localization may improve the diagnostic accuracy of image-guided biopsies and focal ablative therapy [10, 11].

Fluorine-18-labeled prostate-specific membrane antigen (PSMA)-ligand ([^18^F]DCFPyL) is a newer PCa tracer with improved performance in localizing DIL compared to other PET tracers [12–14]. However, one disadvantage of [^18^F]DCFPyL is that it takes a long post-injection time for optimal imaging because of the slow net uptake rate constant (*K*_*i*_ = 0.06–0.20 min^−1^) leading to low tumour-to-background ratio at early times, less than 60 min [15, 16]. Static (single) imaging at least an hour post-injection has been advocated [16–18]. Jansen et al. [16] used dynamic imaging for 60 min and then 90–120-min post-injection and kinetic analysis of acquired dynamic data to derive the tumour-to-blood ratio as a simplified measure of [^18^F]DCFPyL uptake and showed that it is strongly correlated with the net influx rate constant. Shorter-duration (0–22 min) dynamic PET study with [^18^F]DCFPyL has also been reported [15]. This latter study showed that net uptake rate constant (K_i_) and dissociation rate constant (*k*_4_) derived using flow-modified two-tissue compartment (F2TC) kinetics model can robustly detect tumour nodule as identified by prostate sextant biopsy [15]. The F2TC model explicitly takes into account the ingress and egress of [^18^F]DCFPyL into and from tissue via blood flow [15, 19, 20]. Because of this property, we surmise it can reliably subtract the blood background to allow a more accurate evaluation of the uptake from short (< 30 min) data acquisition.

In this study, we compared a short-duration (22 min) dynamic [^18^F]DCFPyL PET scan plus CT Perfusion (CTP) vs 2-h post-injection PET as used in current clinical practice [16–18] to localize DIL using co-registered digital histopathological images as reference. The rationale to limit the acquisition duration of the dynamic scan to 22 min is twofold: 20–30 min is about the longest time for a patient to lie still without discomfort, and we have shown before that kinetic parameters derived from 22 min of dynamic data can detect PCa nodule compared to sextant biopsy even though the method has not been validated against digital pathology of explanted prostates. Dynamic PET and CTP provide complementary molecular and hemodynamic information on DIL and non-DIL prostatic tissue. A secondary goal of this study is to determine whether combining dynamic PET and CTP would better localize DIL than either imaging modality alone.

## Methods

### Study design

This substudy was one arm of a single-centre (Lawson Health Research Institute, London, ON) two-arm clinical study (NCT04009174) to investigate the feasibility of (1) dynamic PET imaging with either [^18^F]FCH (*N* = 25) or [^18^F]DCFPyL (*N* = 27) with CT Perfusion on PCa patients and (2) kinetic analysis of the PET dynamic scans to derive kinetic parameters to localize DIL using co-registered digital histopathological images as reference. The study was approved by the Institutional Research Ethics Board. All participants in this study provided written informed consent. Subjects who had untreated biopsy-proven localized PCa were enrolled in this study. Inclusion criteria were: aged 18 years or older; biopsy-confirmed PCa; suitable for and consenting to radical prostatectomy for treatment. Exclusion criteria were: prior therapy including hormone therapy for PCa, use of 5-alpha reductase inhibitors—finasteride or dutasteride—within 6 months of study date; unable to comply with all pre-operative imaging; prostate size exceeding the dimensions of whole-mount pathology slides; allergy to CT contrast agent; sickle cell disease or other anemias; impaired renal function (estimated GFR < 60 mL/min/1.73 m^2^); residual bladder volume > 150 cc (determined by post-void ultrasound); hip prosthesis and/or vascular graft that are MRI incompatible or other metallic objects within the pelvis; contraindication to MRI, such as a pacemaker or other electronic implants, known metal in orbits and cerebral aneurysm clips. As this substudy was a feasibility study on the two aforementioned goals, sample size was chosen based on availability of radical prostatectomy patients at our site, and no formal calculation was done.

Participating patients in this substudy, from March 2016 to January 2018, underwent the pre-operative imaging session consisting of dynamic [^18^F]DCFPyL PET/CT, CTP, and PET/MR imaging. Dynamic [^18^F]DCFPyL PET/CT was performed first, followed by CTP using a hybrid PET/CT scanner (Discovery VCT, GE Healthcare, Waukesha, WI, USA). At 2-h post-injection of [^18^F]DCFPyL, static PET and MR imaging were performed with a hybrid PET/MR scanner (3 T Biograph mMR, Siemens, Malvern, PA, USA). All patients underwent radical prostatectomy within 6 weeks of all preoperative imaging sessions. The explanted gland with implanted fiducial markers was imaged with T2-weighted (T2w)-MRI and then processed using spatially accurate whole-mount sectioning [21]. Cross sections of the explanted gland spanning the entire mid-prostate were stained with haematoxylin and eosin (H&E) imaging with digital histopathology.

### 22-min dynamic [^18^F]DCFPyL PET/CT imaging

The tracer [^18^F]DCFPyL was purchased from Centre for Probe Development and Commercialization, (Hamilton, ON). Dynamic PET imaging was performed on a Discovery VCT (GE Healthcare, Waukesha, WI, USA) PET/CT scanner. A CT scan was taken with patients lying supine on the patient couch for localization of the prostate and attenuation correction of PET images. The dynamic [^18^F]DCFPyL PET scan covered the whole prostate up to the iliac crest, image-derived arterial time–activity curve required for kinetic analysis of dynamic PET data was acquired from an internal iliac artery to generate parametric maps. Starting at the injection of 325 MBq of [^18^F]DCFPyL as a bolus into an antecubital vein, the dynamic PET scan acquired over 22 min the following number of volumes at each of seven framing intervals: 11 at 10 s, 5 at 20 s, 4 at 40 s, 4 at 60 s, and 4 at 180 s. Each volume comprised of forty-seven 3.27-mm-thick slices. Early time standardized uptake (SUV_Early_) in g/mL was measured as the average of the last four dynamic PET volumes (10–22 min post-injection).

The acquired dynamic volumes were analyzed to generate parametric maps of the whole prostate. We calculated influx rate constant (*K*_1_) in mL/min/g, efflux rate constant (*k*_2_) in min^−1^, binding rate constant (*k*_3_) in min^−1^, dissociation rate constant (*k*_4_) in min^−1^, net uptake rate constant from plasma (*K*_*i*_) in mL/min/g, and distribution volume (DV) in g/mL maps by deconvolving the arterial time–activity curve from tissue time–activity curve using F2TC model [19]. The tissue time–activity curve of each voxel was smoothed by a 3-by-3 mean filter with those from the immediate neighbouring pixels in the same slice.

### CT perfusion (CTP) imaging

Free-breathing CTP scan was performed immediately after the dynamic PET scan without moving the patient. The CTP images were acquired over 3 min using a shuttle mode where two contiguous 4-cm sections of the pelvis covering the prostate and both internal iliac arteries, identified from the CT localization and attenuation correction scan, were alternately scanned starting 6 s after a bolus injection of contrast agent (Isovue 370, Bracco Diagnostic Inc., NJ, USA) at a rate of 3 mL/s and a dosage of 0.7 mL/kg into an antecubital vein. The CTP images were acquired using 5-mm-thick slices, 120 kVp and 50 mAs in two phases: the first phase, at intervals of 2.8 s for first 1 min; the second phase, every 15 s for the next 2 min.

The acquired dynamic CT volumes were co-registered using non-rigid image registration (GE healthcare) to minimize misregistration from breathing motion before functional maps, including blood flow (BF) in mL/min/100 g, blood volume (BV) in mL/100 g, mean transit time (MTT) in second, vessel permeability surface product (PS) in mL/min/100 g, and contrast delay time (T0) in second, and were generated with CT perfusion software (GE Healthcare) [22].

### Two-hour post-injection PET/MR

After the CTP scan, the patient was allowed to rest, lying supine on a couch, in a quiet injection waiting room. At 2-h post-injection, after emptying the bladder, a static PET image of the prostate using limits established with the prior dynamic PET/CT scan was acquired over 15 min on a 3-T Biograph mMR (Siemens, Malvern, PA, USA) hybrid PET/MR scanner [23]. PET images were reconstructed with an ordered subset expectation maximization (OSEM) algorithm with 21 subsets, 3 iterations, and a 4-mm Gaussian filter. Reconstructed resolution was 2.09 × 2.09 × 2.03 mm^3^. MRI was acquired simultaneously with a prostate endorectal receive coil (Medrad), flexible body array (mMR Body), and spine array (mMR Spine). Attenuation correction maps were generated from a 2-point Dixon acquisition segmented for water, fat, and air. T2-weighted MRI included 2D transverse, sagittal, and coronal acquisitions as well as a 3D acquisition. Two-hour post-injection SUV (SUV_Late_) maps in g/mL were measured from the acquired images.

### Image registrations and analysis

After surgical resection, the prostate specimen was placed in 10% buffered formalin and marked with fiducial markers [21]. The specimen was then temporarily placed in a perfluoropolyether oil (Christo-Lube, Lubrication Technology, Franklin Furnace, OH, USA)-filled container for imaging. Ex vivo T2w-MRI was performed with a 3-T Discovery MR750 scanner (GE Healthcare, Waukesha, WI, USA). The oil provided a black background to minimize boundary artefacts in the MR images of the explanted prostate. After fixing in formalin for 48 h, the specimen was processed *as per* the standard pathology grossing protocol at our institution and submitted for routine processing, paraffin embedding, and reporting. Whole-mount 4-μm-thick H&E-stained histology cross sections spanning the entire midprostate were then digitized for contouring and histopathological scoring by pathologists.

**Fig. 1:**
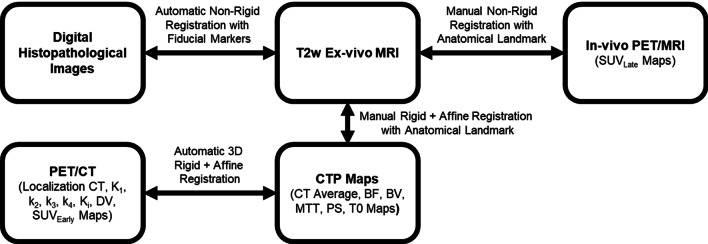
Registration pipeline. *CT* Computed tomography, *T2w* transverse relaxation time weighted, *PET* positron emission tomography, *MR* magnetic resonance, *BF* blood flow, *BV* blood volume, *MTT* mean transit time, *PS* vessel permeability surface product, *T*_*o*_ contrast delay time, *K*_*1*_ influx rate constant, *k*_*2*_ efflux rate constant, *k*_*3*_ binding rate constant, *k*_*4*_ dissociation rate constant, *K*_*i*_ net uptake rate constant, *DV* distribution volume, *SUV* standardized uptake value

In order to correlate all PET, CT, and MR maps to cancerous nodules identified in digital histopathological images of the explanted prostate, a registration pipeline, as shown in Fig. 1, was developed. The ex vivo T2w-MR was used as the bridge to co-register ex vivo digital histopathology to in vivo PET, CT, and MR. All these images were registered using a non-rigid or rigid-affine registration to compensate for any shrinkage of the explanted prostate. Histology and ex vivo MR co-registration was done using automatic non-rigid registration with the marked fiducial markers and anatomical structures/landmarks [21]. Co-registration of ex vivo MRI and in vivo PET/MRI was done using user-identified homologous anatomical landmarks (i.e. benign prostatic hyperplasia nodules, urethra, calcifications, etc.) via a manual thin plate spline non-rigid registration [11]. Co-registration of CTP average map and ex vivo T2w-MRI was done using anatomical landmarks via manual rigid-affine registration in ITK-SNAP (www.itk-snap.org) [24]. PET-CTP co-registration was achieved by registering the CT images acquired for attenuation correction of dynamic PET and CTP average maps with the automatic 3D rigid-affine registration module—General Registration—in 3D slicer (www.slicer.org) [25]. After co-registration of all PET and CTP parametric maps with digital histopathological images, regions of interest were drawn on the latter images, to encompass the DIL and the entire prostate outside the DIL (non-DIL) by pathologists (contoured by MG and verified by MM and JAG). The DIL was identified as the largest cancerous lesion within the histopathological images. These ROIs were superimposed on all corresponding PET and CTP maps to generate voxel and volumetric data for DIL and non-DIL prostatic tissue. Additionally, anatomical features such as calcifications and urethra in the co-registered PET, MR, and CTP images were compared to the digital histopathological images to determine the error of registration which was found to be between 3 mm (CT and MR) and 5.5 mm (one PET voxel).

### Statistical analysis

Statistical analyses were performed in SPSS Statistics 27 (IBM Analytics, Armonk NY), using two-sided statistical testing with significance accepted at *P* < 0.05. Descriptive statistics were presented as mean and SD or as median and range (min to max). Differences between DIL and non-DIL imaging parameters in DIL and non-DIL ROIs were compared using the Wilcoxon signed-rank test with Bonferroni correction for multiple comparisons. Voxel-wise multivariable logistic regression with backward elimination was used to determine the most accurate model of [^18^F]DCFPyL PET and CTP parameters from a subset, each of which attained *P* < 0.05 in univariable Wilcoxon signed-rank testing, to distinguish DIL from non-DIL tissue. We also performed leave-one-patient-out cross-validation to validate the selected logistic regression model for voxel-wise analysis and reported the average and standard error of error rate (ER), false-positive rate (FPR) and false-negative rate (FNR), area under the receiver operating characteristic curve (AUC) of all folds.

## Results

### Patients

**Fig. 2:**
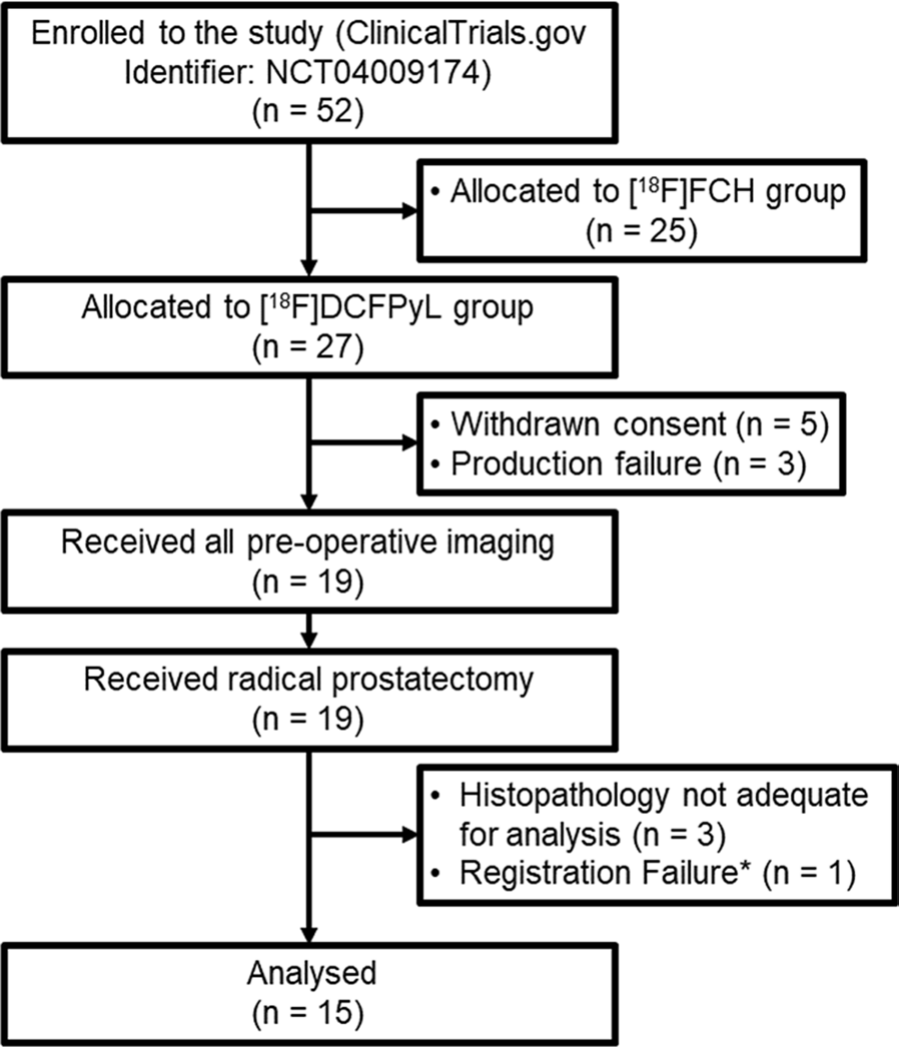
Flow diagram showing patient enrolment. *Registration failed because the in vivo prostate was significantly deformed relative to explanted prostate due to gas in the rectum. *FCH* Fluorocholine, *DCFPyL* 2-(3-{1-Carboxy-5-[(6-[^18^F]fluoro-pyridine-3-carbonyl)-amino]-pentyl}-ureido)-pentanedioic acid

Twenty-seven male patients with biopsy-proven PCa were enrolled in the [^18^F]DCFPyL arm of a clinical trial (NCT04009174, Fig. 2). A subset of 19 patients completed both imaging sessions—[^18^F]DCFPyL PET/CT, CTP, and [^18^F]DCFPyL PET/MR and also underwent radical prostatectomy. Of those, 15 had analyzable digital histopathological images from the mid-section of the gland. Figure 2 shows the patient flow through the study. The detailed clinical–pathological characteristics of 15 patients (median age 63 year; range 53–68 year) who were included in this study are listed in Table 1.

**Table 1:** Clinical–pathological characteristics of patients and tumours (*n* = 15)

Age [y]	62.1 ± 4.1 (53–68)*
Weight [kg]	88.8 ± 11.4 (65–109)*
Height [cm]	177.4 ± 6.1 (170–191)*
PSA [ng/mL]	9.4 ± 6.3 (3.5–25.5)*
Injected [^18^F]DCFPyL activity [MBq]	312.4 ± 15.9 (280.8–348.2)*
Histology—*n*(%)	
Adenocarcinoma	15 (100)
pT Stage—*n*(%)	
T2c	7 (46.7)
T3a	7 (46.7)
T3b	1 (6.7)
pN Stage—*n*(%)	
N0	15 (100)
N1	0 (0)
NX	0 (0)
pM Stage—*n*(%)	
M0	15 (100)
M1	0 (0)
MX	0 (0)
Gleason score of DIL—*n*(%)	
6 (3 + 3)	1 (6.7)
7 (3 + 4)	6 (40.0)
7 (4 + 3)	5 (33.3)
9 (5 + 4)	3 (20.0)

Data are the number of patients with the percentage in parentheses unless otherwise indicated

*DCFPyL* 2-(3-{1-Carboxy-5-[(6-[^18^F]fluoro-pyridine-3-carbonyl)-amino]-pentyl}-ureido)-pentanedioic acid, *PSA* prostate-specific antigen, *pT* primary tumour, *pN* lymph node, *pM* distant metastasis, *DIL* dominant intraprostatic lesion

*Data are means ± standard deviation, with the range in parentheses

### Descriptive statistics and parameter selection

There were 13 parametric maps per patient generated from dynamic [^18^F]DCFPyL PET, CTP, and 2-h post-injection PET/MR. Total of 49,254 voxels were analyzed in DIL and non-DIL ROIs. Using a cut-off of *P* < 0.05 after Bonferroni correction for multiple comparison for selecting potentially significant variables, Wilcoxon singed-rank test revealed that the following parameters distinguished DIL versus non-DIL tissue—BF (median 56.58 vs. 46.43 mL/min/100 g, *P* < 0.01), BV (median 8.92 vs. 5.83 mL/100 g, *P* < 0.01), MTT (median 8.94 vs. 8.07 s, *P* < 0.01), PS (median 24.39 vs. 19.44 mL/min/100 g, *P* < 0.05), *K*_1_ (median 0.236 vs. 0.187 mL/min/g, *P* < 0.01), *k*_4_ (median 0.085 vs. 0.130 min^−1^, *P* < 0.05), *K*_*i*_ (median 0.055 vs. 0.036 mL/min/g, *P* < 0.05), DV (median 2.29 vs. 1.17 mL/g, *P* < 0.001), SUV_Early_ (median 2.53 vs. 1.78 g/mL, *P* < 0.001), and SUV_Late_ (median 1.55 vs. 0.67 g/mL, *P* < 0.001).

Among these parameters from PET and CTP, the combination of K_i_ and k_4_ was the most accurate model to distinguish DIL from non-DIL voxel using multivariable logistic regression with backward elimination (*P* < 0.001). If only significant CTP parameters were used, the BF and MTT model was the most accurate model (*P* < 0.001).

### Cross-validation and diagnostic performance

**Fig. 3:**
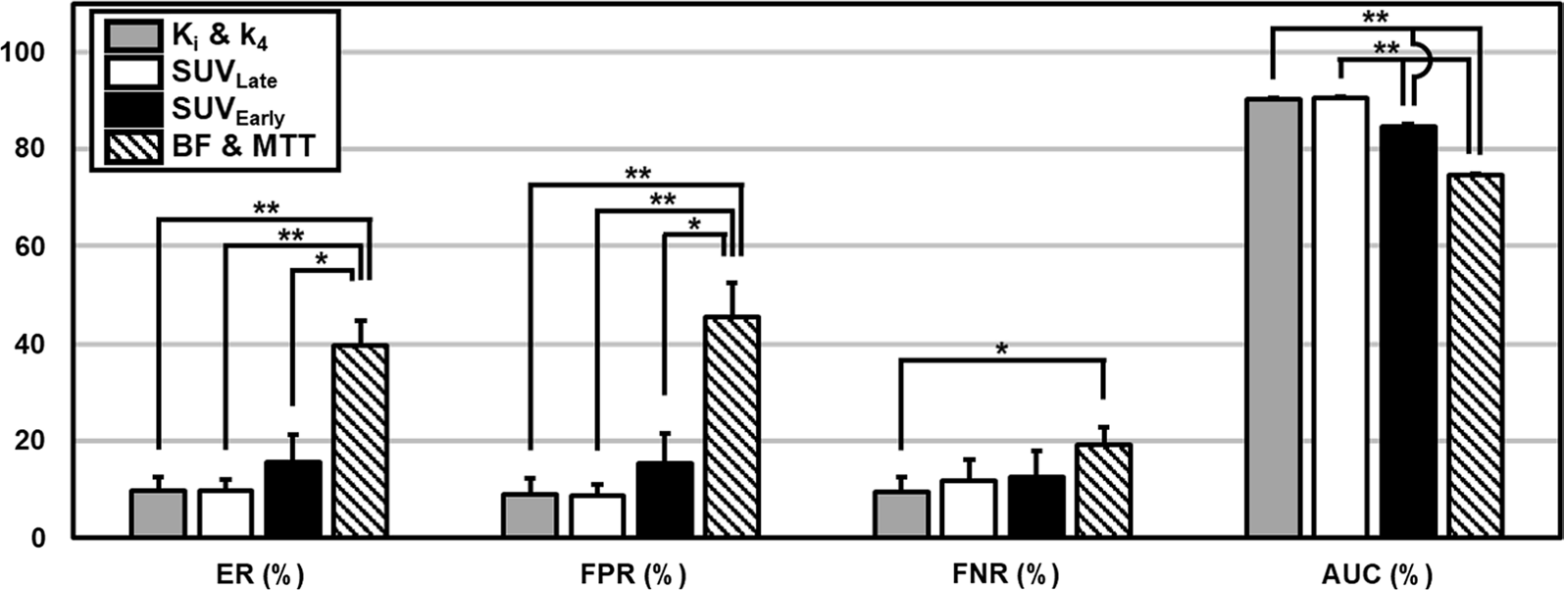
Comparison of leave-one-patient-out cross-validation metrics of the different DIL detection models—error rate (ER), false-positive rate (FPR), false-negative rate (FNR), and AUC. Error bars are the standard deviation of mean. A significant difference (*P* < 0.05) is marked with asterisk (*) for *P* < 0.05 and double asterisk (**) for *P* < 0.001. *K*_*i*_ Net uptake rate constant, *k*_*4*_ dissociation rate constant, *SUV*_*Late*_ 2-h post-injection standardized uptake value, *SUV*_*Early*_ 10-min post-injection standardized uptake value, *BF* blood flow, *MTT* mean transit time, *ER* error rate, *FPR* false-positive rate, *FNR* false-negative rate, *AUC* area under the curve of receiver operating characteristic curve, *DIL* dominant intraprostatic lesion

Four DIL detection models—*K*_*i*_ and *k*_4_; BF and MTT; SUV_Early_; and SUV_Late_—were validated using leave-one-patient-out cross-validation. The ER, FPR, FNR, and AUC obtained for each model are shown in Fig. 3. *K*_*i*_ and *k*_4_ were the most accurate and stable model to detect DIL. It had ER of 9.9 ± 2.8%, FPR of 9.1 ± 3.1%, FNR of 9.6 ± 3.0%, and AUC of 90.4 ± 0.2%. BF and MTT model showed ER, FPR, FNR, and AUC of 39.6 ± 5.1%, 45.5 ± 6.8%, 19.3 ± 3.6%, 74.8 ± 0.2%, respectively. SUV_Early_ model showed ER, FPR, FNR, and AUC of 15.7 ± 5.6%, 15.3 ± 6.2%, and 12.7 ± 5.3%, 84.7 ± 0.5%, while for SUV_Late_, ER, FPR, FNR, and AUC of 9.8 ± 2.3%, 8.7 ± 2.4%, 11.9 ± 4.3%, 90.5 ± 0.3%. AUC of *K*_*i*_ and *k*_4_ versus SUV_Late_ was not significantly different, but both were better than SUV_Early_ and BF and MTT. FNR was similar among all models except that BF and MTT were significantly worse than K_i_ and k_4_. For FPR and ER, only BF and MTT were significantly worse than the other models.

Dice similarity coefficient which gauged the similarity in size and location between the DIL detected by each model against that delineated by pathologists (MM,MG) on the digital histopathological images was 0.8 ± 0.1, 0.4 ± 0.1, 0.7 ± 0.1, and 0.7 ± 0.1, respectively, for *K*_*i*_ and *k*_4_ model, BF and MTT model, SUV_Early_ and SUV_Late_.

## Discussion

In this study, we investigated using dynamic [^18^F]DCFPyL PET kinetic parameters, CTP parameters, and SUV_Late_ to localize DIL in 15 patients and validated the developed method against digital histopathological images. Our analysis showed that *K*_*i*_ (net uptake rate constant of [^18^F]DCFPyL from plasma) and k_4_ (dissociation rate constant of [^18^F]DCFPyL after binding to PSMA on PCa cells) model from 22-min dynamic PET was the more accurate and stable model to detect and localize DIL with excellent diagnostic performance versus digital histopathology as validated using leave-one-patient-out cross-validation (Fig. 3).

Figure 3 shows three important aspects of the results: (1) SUV_Late_ showed better DIL detection results than SUV_Early_ (AUC 90.5 ± 0.3% vs. 84.7 ± 0.5%, *P* < 0.001), (2) *K*_*i*_ and *k*_4_ model also showed better results than SUV_Early_ (AUC 90.4 ± 0.2% vs. 84.7 ± 0.5%, *P* < 0.001), and 3. K_i_ and *k*_4_ model from 22-min dynamic scan achieved comparable results to the 2-h post-injection SUV_Late_ (AUC 90.4 ± 0.2% vs. 90.5 ± 0.3%, *P* > 0.05). These results agree with the literature recommendation that if SUV is used to detect PCa, the imaging has to be performed at > 1 h post-injection [16–18]. Furthermore, the K_i_ and k_4_ model achieved the best dice similarity coefficient and FNR against SUV_Early_ and SUV_Late_. This is significant for image-guided biopsies and local radiation therapy (e.g. focal ablative therapy or radiation boost) because imaging modality with the lowest tumour omission is preferred. In terms of avoiding overtreatment of non-tumour tissue, the *K*_*i*_ and *k*_4_ model and SUV_Late_ had the same FPR which was smaller than SUV_Early_.

**Fig. 4:**
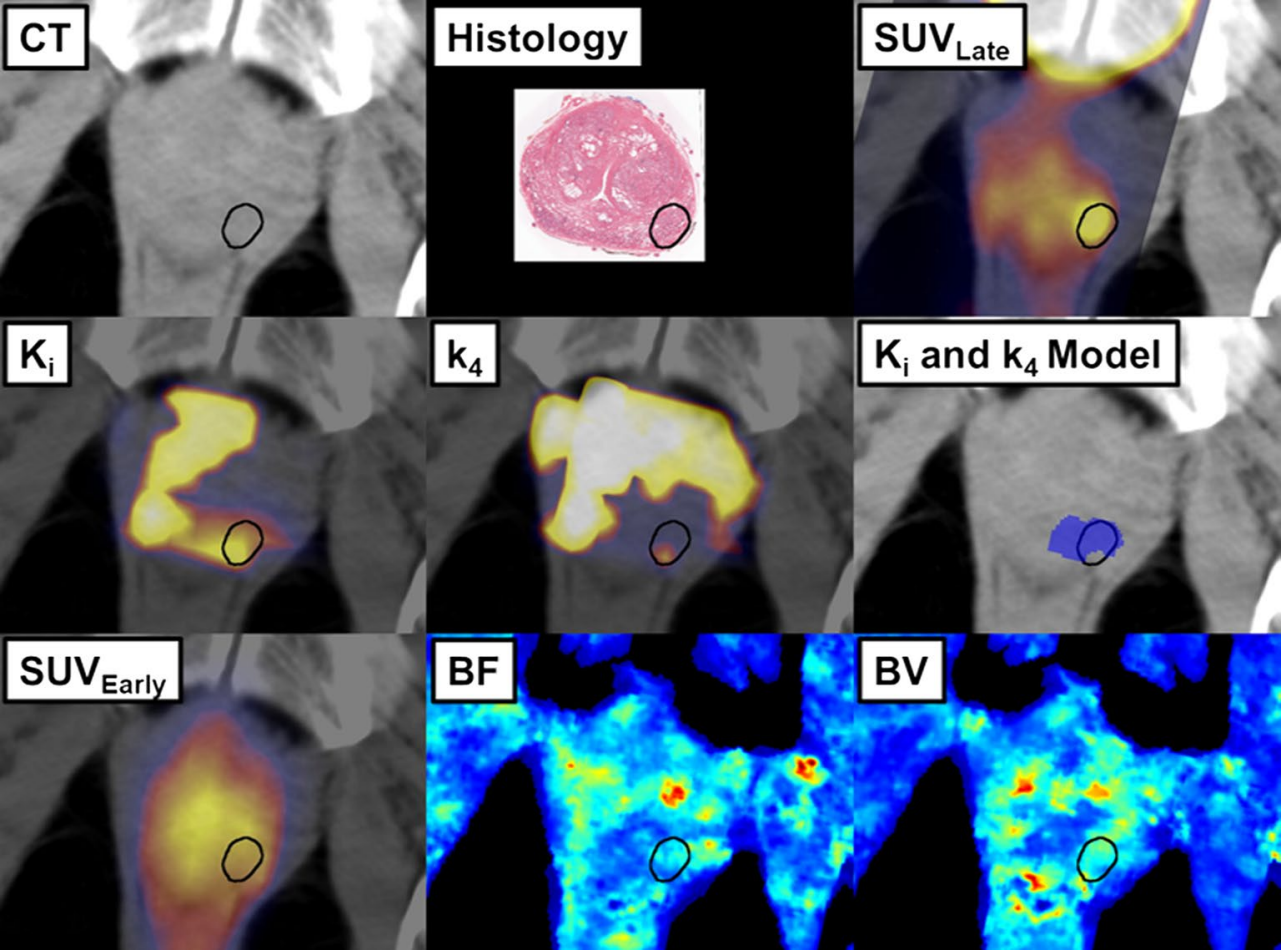
Example of the co-registered in vivo images and parametric maps. The location of DIL is outlined in black. *CT* Computed tomography, *PET* positron emission tomography, *SUV*_*Late*_ 2-h post-injection standardized uptake value, *K*_*i*_ net uptake rate constant, *k*_*4*_ dissociation rate constant, *SUV*_*Early*_ 10-min post-injection standardized uptake value, *BF* blood flow, *BV* blood volume, *DIL* dominant intraprostatic lesion

Figure 4 shows qualitatively the distinction of DIL from non-DIL tissue using the different parametric maps in a patient. It is noteworthy that the linear classifier from the *K*_*i*_ and *k*_4_ model shows that DIL had high net uptake rate constant (*K*_*i*_) of the tracer and low tracer dissociation rate constant from PSMA once bound (*k*_4_). This shows that even with favourable kinetics, SUV acquired at a short time post-injection, as was SUV_Early_ in this study, cannot reliably differentiate DIL from non-DIL tissue because tissue uptake was dominated by high BF and BV (see Fig. 4) or the high blood background. SUV measured with a single static image at a fixed time after injection cannot differentiate the different processes driving the tracer uptake including: tracer delivery to and washout from tissue via blood flow, bidirectional exchange between vessels and tissue, and binding to and dissociation from the target. The F2TC model used in the kinetic analysis of dynamic [^18^F]DCFPyL data in this study was able to separate the different uptake processes, particularly removing the blood background, hence provided a better characterization of the different uptake of the tracer in DIL vs. non-DIL tissue at early times post-injection. Even though our short-duration (22 min) study may result in biased estimates of the uptake parameters: *K*_*i*_, *k*_3_, *k*_4_, and DV, nevertheless two of the parameters, *K*_*i*_ and *k*_4_, were able to reliably distinguish between DIL and non-DIL with performance non-inferior to that of SUV_Late_. From the clinical perspective, the short duration would increase the throughput of [^18^F]DCFPyL studies to detect and localize DIL.

In this study, we showed that among the four clinical methods for identifying DIL, dynamic PET-derived *K*_*i*_ and *k*_4_ and SUV_Late_ are equivalent and better than SUV_Early_ or CTP and CTP is the worst. In comparing *K*_*i*_ and *k*_4_ versus SUV_Late_, the former has the advantage that delayed (2-h post) imaging is not required simplifying scheduling and increasing convenience to patients. We also found that CTP parameters did not add to the diagnostic performance of PET in distinguishing DIL from non-DIL tissue (results not shown). A possible explanation is that blood flow is already subsumed in the net uptake rate constant from plasma (*K*_*i*_). Using just CTP parameters, the BF and MTT model showed AUC of 74.8 ± 0.2%, which was lower than the other models based on [^18^F]DCFPyL. Nevertheless, these results were still comparable to the AUC of multi-parametric MR (mp-MRI), which ranged from 68 to 77% [26–28]. An important advantage of CT perfusion is that it can be easily incorporated into current clinical CT scanning protocols used in diagnosis and treatment follow-up by adding only a couple of minutes in scanning time and being less expensive than either mp-MRI or PET. Additionally, CTP imaging lends itself to integration with existing CT planning processes for radiation treatment, potentially enabling differential tumour volume boosting [29].

This study has limitations. First, the number of enrolled patients in this study was small and was further reduced by various reasons as discussed in ‘Results’ raising the possibility of selection bias. As such, the results obtained must be interpreted with caution but warrant confirmation with another study with a larger number of patients. Second, we used binomial logistic regression limiting our detection method to two tissue types—DIL and non-DIL. A trinomial logistic regression would allow separate of three tissue types—DIL, non-DIL cancer nodules, and benign prostatic tissue. The small number of patients available precluded a reliable investigation of this possibility. However, if the goal is to identify DIL for boosting the radiation dose and more precise targeting of biopsy, a two-tissue type separation method would be sufficient for this purpose.

## Conclusion

Using kinetic analysis to remove blood background, we showed that the kinetic parameters *K*_*i*_ and *k*_4_ derived from a 22-min dynamic [^18^F]DCFPyL PET can detect DIL as accurately as SUV measured at 2-h post-injection. As the method can improve the throughput and logistics of using [^18^F]DCFPyL PET for the management of prostate cancer, further study with larger sample size is warranted.

## Data Availability

All reasonable request for research data from this study will be granted and provided it is used for academic purposes.
